# Flap adhesion and effect on postoperative complication rates using Tissuglu^®^ in mastectomy patients

**DOI:** 10.1007/s12282-015-0591-1

**Published:** 2015-02-10

**Authors:** Christian Eichler, Petra Fischer, Axel Sauerwald, Faten Dahdouh, Mathias Warm

**Affiliations:** 1 Department of Gynecology and Obstetrics, Holweide Hospital, Cologne, Germany; 2 Brustzentrum, Krankenhaus Holweide, Neufelder Strasse 32, 51067 Köln, Germany; 3 Department of Gynecology and Obstetrics, Hospital Düren GmbH, Düren, Germany; 4 Department of Gynecology and Obstetrics, University of Cologne, Cologne, Germany

**Keywords:** Breast cancer, Mastectomy, Seroma, Prevention, TissuGlu, Drain, Time to drain removal

## Abstract

**Introduction:**

Post-mastectomy seroma and related complications are common problems in modern oncological surgery. Occurrence rates of up to 59 % have been reported in literature. High-risk patients, that is, those who have undergone previous surgeries, present with a high body mass index, have had radiation or chemotherapy, present a particular challenge. Noninvasive measures such as fibrin-based sealants have thus far not been able to effectively reduce complications associated with fluid accumulation. A recent study using a lysine-derived urethane adhesive named TissuGlu^®^ however, showed promising results in patients after abdominoplasty.

**Methods:**

32 consecutively recruited patients received a mastectomy using a gold standard mastectomy technique as well as TissuGlu^®^ flap fixation. A control group of 173 patients, having received a gold standard mastectomy-only, was analyzed retrospectively, totaling 205 patients. Primary endpoints were post-discharge seroma formation and revision surgery/re-hospitalization. Secondary endpoints were initial seroma volume, postoperative pain, hematoma formation and day of drain removal.

**Results:**

No significant difference in seroma formation was demonstrated. The revision surgery/re-hospitalization rate was reduced from 6.9 to 0 %, though this did not reach significance. Significant improvement could be shown in the TissuGlu^®^ group regarding time to drain removal (17 % decrease), and hematoma formation (14 % decrease). No difference was shown in postoperative pain.

**Conclusion:**

Although patient numbers are still small, advantages in revision surgery/re-hospitalization rate, hematoma formation as well as time to drain removal was shown for the TissuGlu^®^ group.

**Clinical question/level of evidence:**

Therapeutic, IV.

## Introduction

Post-mastectomy seroma and its associated complications remain problematic in modern oncological surgery [1, 2]. The most common approach for preventing seroma is the placement of surgical drains within the wound area, though the efficacy of this approach is not universally acknowledged and the problem persists in spite of the use of drains [1]. Other approaches have been evaluated to reduce the incidence of complications associated with seroma formation, but a consensus on the best solution remains elusive. Recent studies aimed at the reduction or elimination of dead space have shown some promise in reducing these complications in other surgical techniques. Two options include the use of progressive tension or quilting suture techniques and the less invasive application of a lysine-derived urethane adhesive (TissuGlu^®^, Cohera Medical, Pittsburgh, PA, USA) [3–7]. Our group already demonstrated the use of TissuGlu^®^ in the successful resolution of a persistent post-mastectomy seroma [8]. While a plethora of literature is available on the benefits of post-surgical drain placement, due to the persistence of the problem some authors argue that drain placement does not represent a satisfactory solution and may not be necessary at all [9]. Nonetheless, most surgeons still place drains in the wound area. To safely evaluate a new option for reduction of postoperative complications in mastectomy surgery, however, this method should be tested against the surgical gold standard, which is a mastectomy with drain placement. Risk factors such as radiation treatment, chemotherapy, smoking habits, increased age and obesity are described in literature [4, 5, 10–12] and are outside of the control of the treating physician. Eliminating dead space in the wound area may, however, be successfully achieved by the surgeon. An interesting question is thus to evaluate if mastectomies performed using a surgical gold standard, including a suction drain, may yield better results when this adhesive is added.

## Patients and methods

A total of 205 breast cancer patients were analyzed in this study. For patients who received a bilateral mastectomy, both sides were evaluated individually. This was the case once in the TissuGlu^®^ group and seven times in the control group. 173 data sets were investigated retrospectively with regard to age, smoking habits, body mass index (BMI) as well as prior chemotherapy or previous radiation treatment. The specific oncological character of each patient will not be listed in this analysis since histology, grading, staging, hormone receptor status, etc., does not impact surgical outcome [13]. The decision to perform unilateral or bilateral mastectomy was always derived from a tumor board. Thus, gold standards in tumor therapy were adhered to. All data were collected at the breast cancer center of the Municipal Hospital Holweide, Cologne, Germany between 2010 until 2013. The TissuGlu^®^ group consisted of consecutively enrolled patients in the year 2013. Primary endpoints were postoperative complication rates, i.e. seroma formation and severe adverse events. The latter was considered to have occurred if either re-hospitalization within 6-month post-surgery became necessary due to wound healing complications (e.g. infection, deep hematoma, wound dehiscence) or in the case of persistent seroma requiring treatment. Secondary endpoints were post-surgical superficial hematoma, day of drain removal, postoperative pain as well as total volume drained.

### Surgery and post-surgical management

**Fig. 1:**
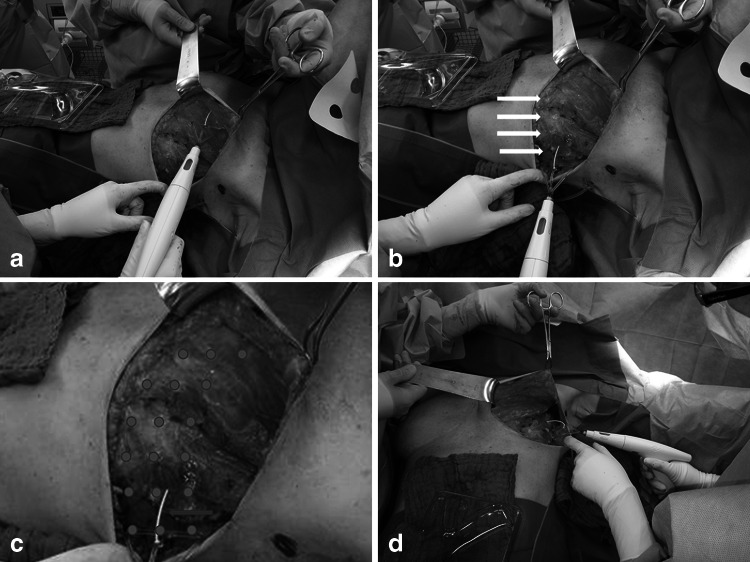
Mastectomy with sentinel lymph node dissection. Shown is the surgical situs during TissuGlu^®^ (TG) application. **a** the medial application of adhesive droplets using the provided TG applicator. Droplet distribution was performed in a medial to lateral manner (**b**). The *white arrows* indicate TG droplets. **c** Schematically depicts droplet spacing on the wound surface. The *red arrow* indicates the droplet spacer. **d** The wound cavity from a caudal angle

Naturally, several surgical approaches were available. Mastectomies were performed with or without axillary lymph node dissection, possibly in sentinel technique. Drain placement is common practice in either situation. If patients received an axillary lymph node dissection along with a mastectomy, drains would be placed in both areas. Analysis of the seroma formation focused on the mastectomy area only. All surgeries were performed by the same, experienced surgical team. The surgical procedure was also the same for both groups, although the surgical adhesive was added in the TG group. Figure 1 shows the TissuGlu^®^ application. Equal adhesive droplet placement is ensured using an adhesive applicator. Following wound closure, the surface is compressed and immobilized. Compression was applied by an elastic bandage, wrapped around the thorax, which was removed after 24 h. The compression regime was the same for all mastectomy patients. Drains were placed in all surgical sites, volume drained was recorded and drains were removed when less than 30 ml/24 h fluid was recorded, with the exception of the first postoperative day. Drains remained in place for at least 24 h as standard in-house protocols demanded. All drains were non-suction drains.

Post-surgical follow-up for the TG group included regular clinician wound inspection and an additional pre-discharge inspection as well as a 14-day follow-up. Seroma formation after drain removal was detected using ultrasound and palpation. A bias in favor of the control group had to be accepted, since only clinically relevant seroma, i.e. painful, infected, etc., was recorded for these patients, as they would only revisit our hospital when such problems occurred. Regular 14-day follow-up was provided in the control group through their family practitioners. The TG group, on the other hand, was more closely monitored; seroma formation was recorded even if not clinically relevant. To answer the central question of this paper as conservatively as possible, we accepted this bias towards the control group so as to not skew results in favor of the TissuGlu^®^ approach.

### Informed consent

Written informed consent was obtained from all patients for publication of this manuscript. A copy of the written consent is available for review by the Editor-in-Chief of this journal. This study was conducted in accordance with institutional review board standard operating procedures.

### Statistics

Statistical analysis was performed using the Vassar Stats^®^ (Vassar College, Poughkeepsie, NY, USA) statistics program. Pearson’s Chi squared tests and *t* tests were used to evaluate significances when appropriate.

## Results

Patient age was similar in both groups. The control group had a patient age of 62 (±14) years while the consecutively recruited TissuGlu^®^ group had an average age of 67 (±13) years (*p* > 0.05). Table 1 shows a summary of all results. BMI, prior chemotherapy and prior radiation treatment did not differ significantly. The average BMI was 27 (±7) and 26 (±7) in the control and TG, respectively. Prior radiation therapy was received by approximately 17 % of the control group patients, and 16 % of the TG group. Smoking habits differed significantly in favor of the TG group, where none of the patients listed a smoking habit, in comparison to the control group where approximately 14 % smokers were included. With exception of smoking habits, adequate comparability is thus given.

**Table 1:** Summary of seroma risk factors, primary and secondary endpoints

	Control		TissuGlu^®^		*p* value
	Total	%	Total	%	
*n*	173		32		
Age (years)	62 (±14)		67 (±13)		0.094
BMI	27 (±7)		26 (±7)		0.889
Previous chemotherapy	44	25.4	4	12.5	0.174
Radiation	30	17.3	5	15.6	0.807
Nicotine	24	13.9	0		0.007
Superficial post-surgical hematoma	29	16.8	1	3.1	0.045
Post-surgical pain (1–10)	1.5 (±1)		2,7 (±0, 8)		0.787
Day of drain removal	4.2 (±1.8)		3,5 (±1, 5)		0.008
Total volume	191 (±160)		152 (±116)		0.08
Follow-up seroma	27	15.6	8	27.6	0.06
Adverse events (revision/infection)	12	6.9	0		0.22

Primary endpoint results showed that approximately 16 % of the control group and 28 % of the TG group developed a postoperative seroma; this difference not being significant (*p* > 0.05). The clinical relevance of this fluid accumulation should, however, be questioned since 7 % of the control group patients required revision surgeries or re-hospitalization and i.v. antibiotic treatment (3 cases) due to infection, whereas none of the TissuGlu^®^ patients required re-hospitalization at all.

The secondary endpoints showed that superficial postoperative hematoma presented in 17 % of the control group versus 3 % in the TG group (*p* < 0.05). No difference was shown in postoperative pain. Time to drain removal also differed significantly in favor of the TG group with 4.2 (±1.8) control groups vs. 3.5 (±1.5) TG days (*p* < 0.05). The mean total amount of fluid collected per patient was also lower the TG group, although no statistical significance could be established here. Table 2 shows a subgroup analysis. Inter-group comparability is given. Distributions do not vary significantly for mastectomy-only, mastectomy plus SNL biopsy and mastectomy plus complete axillary dissection subsets. Results show adverse events to be evenly distributed within the control group. No adverse events are found in the TG group. Seroma formation occurs with the same frequency in mastectomy-only (48 %) and mastectomy-plus axillary dissection (44 %) subsets within the control group. This is not the case in the TG group. Here, 88 % of post-surgical seroma is found in the mastectomy group. The difference in distribution is most likely due to small sample size.

**Table 2:** Subgroup analysis: intergroup distributions do not differ significantly with respect to number of mastectomies. SNL biopsy and axillary dissections. Post-surgical seroma formation occurred more often in the mastectomy group for TG

	Distribution (% of total)	Adverse event (% of total)	Seroma (% of total)
Control			
Mastectomy	63 (36 %)	5 (38 %)	13 (48 %)
Axilla	79 (46 %)	4 (31 %)	12 (44 %)
SNL	31 (18 %)	4 (31 %)	2 (7 %)
Total	173	13	27
TissuGlu^®^			
Mastectomy	14 (44 %)	–	7 (88 %)
Axilla	11 (34 %)	–	–
SNL	7 (22 %)	–	1 (13 %)
Total	32	–	8
*p*	0.223		>0.001

## Discussion

Study group homogeneity needs to be established before comparing the two study arms. As patient groups do not differ in the age, previous radiation and chemo therapy nor BMI [14], comparison is possible. This was paramount as these are the most common risk factors for developing seroma or wound healing complication [4, 5, 9–11]. Smoking habits favored the TG group and future patient recruitment will have to take this fact into account. Only 3 of the 12 patients with an adverse event in the control group were smokers.

### Primary endpoints

One of the primary endpoints seems to favor the control group, since approximately 16 % of the control group developed a post-hospital discharge follow-up seroma vs. 28 % of the TissuGlu^®^ group (*p* > 0.05). As these results do not differ significantly, no definite conclusion may be drawn at this point. Nonetheless, it is important to discuss this issue. We may have to accept the possibility that the use of TG may lead to an increase in postsurgical seroma. This has not been shown or reported in previous literature and should be investigated in future trials with larger numbers. In addition, a bias in favor of the control group had to be accepted due to the retrospective nature of the control group analysis. As mentioned above, patients of the control group did not participate in mandatory follow-up visits in our in-house standard of care. Only clinically relevant seroma, i.e. patients presenting with pain, discomfort, etc., was recorded, as these patients returned seeking intervention. Clinically non-relevant seroma, i.e. painless, small volume seroma, could not be documented. Since post-surgical follow-up of the TG groups included several clinical inspections as well as ultrasound analysis of the immediate post-drain removal situation, non-clinically relevant seroma formation was in turn documented in this group. While a comparison between the two groups may nonetheless be drawn, this issue will be addressed in a follow-up head-to-head prospective study.

Seven percent (*n* = 12) of the control group experienced a severe adverse event as defined above, and 0 % of the TissuGlu^®^ showed such complications. This supports the hypothesis above that seroma documented in the TG group for the primary endpoint may have been clinically non-relevant. Unfortunately, a significance level could not be achieved due to small sample number (*p* > 0.05).

### Secondary endpoints

A recent study investigating a new 50 ml/24 h fluid production limit as an indicator for drain removal has demonstrated this volume to be an adequate and safe parameter for drain removal [15]. Our study used a limit of 30 ml/24 h which may be viewed as a more challenging approach to secondary endpoint analysis. Overall, patient satisfaction (subjectively) was high in the TissuGlu^®^ group due to earlier drain removal: 3.5 days TG versus 4.2 days in the control group (*p* < 0.05). This represents a significant decrease in time to drain removal by 17 %. Although the relevance of this may be theoretical in nature, the simple fact remains that drains may be removed earlier without increasing postoperative complication rates. Although patient satisfaction could not be retrospectively quantified, drain removal almost always results in a decrease in local discomfort, which in turn increases patient satisfaction. Future analyses will, therefore, attempt to quantify this aspect. Nonetheless, we are presented with an option for earlier drain removal when using TG, without significantly increasing the post-surgical complication rates [15–17].

Interestingly, post-surgical hematoma was significantly lower in the TissuGlu ^®^ group (3 %) in comparison to the control group (29 %) (*p* < 0.05). This issue will also have to be addressed in a future prospective study since no hemostatic properties of TissuGlu^®^ are known. Since postoperative care was the same, with the exception of the follow-up visits, this phenomenon could not be explained. One may, however, speculate that by effectively eliminating dead space, surface to surface abrasion may be minimized.

## Conclusion

This analysis shows that introducing TissuGlu^®^ into a mastectomy area is a useful tool for improving overall surgical outcome. Non-inferiority could be shown for both primary endpoints. Follow-up seroma formation and postoperative adverse events did not differ significantly. A strong trend towards lower postoperative complication rates in the TissuGlu group was shown. Secondary endpoints showed significant improvement in the TissuGlu^®^ group with a 17 % decrease in time to drain removal as well as a 14 % decrease in postoperative hematoma formation.
